# Effect of Glucagon‐Like Peptide 1 Receptor Agonists on Obstructive Sleep Apnea

**DOI:** 10.1002/osp4.70090

**Published:** 2025-08-22

**Authors:** Bei‐Bei Qian, Yu‐Jie Huang, Cai‐Feng Yan, Shang‐Yong Feng, Dun‐Min She

**Affiliations:** ^1^ Northern Jiangsu People's Hospital Northern Jiangsu People's Hospital Affiliated to Yangzhou University Yangzhou Jiangsu China; ^2^ Huaian Cancer Hospital Huaian Jiangsu China

**Keywords:** GLP‐1R agonists, Mendelian randomization, obesity, obstructive sleep apnea, type 2 diabetes

## Abstract

**Background and Aim:**

Glucagon‐like peptide‐1 receptor (GLP‐1R) agonists are well‐established therapies for obesity and type 2 diabetes mellitus (T2DM). Emerging evidence also suggests their potential role in managing obstructive sleep apnea (OSA). This study aimed to investigate the association between GLP‐1R agonists and OSA using genetic evidence.

**Methods:**

Cis‐expression quantitative trait loci (cis‐eQTLs) associated with the *GLP1R* gene were identified and used as genetic proxies for GLP‐1R agonist exposure. To validate the selected genetic instruments, positive control analyses were conducted for T2DM and body mass index (BMI). Mendelian randomization was employed to evaluate the effect of genetically proxied GLP‐1R agonists on OSA. OSA data were obtained from FinnGen Release 11 (R11), comprising 50,200 cases and 401,484 controls of European ancestry. The inverse variance weighted (IVW) method served as the primary analytical approach, supplemented by heterogeneity tests and sensitivity analyses.

**Results:**

IVW analysis showed that genetically predicted GLP‐1R agonist exposure was associated with a reduction in BMI (*β* = −0.063, 95% confidence interval [CI]: −0.10 to −0.03, *p* = 8.43 × 10^−4^) and a decreased risk of T2DM (odds ratio [OR] = 0.80, 95% CI: 0.65 to 0.98, *p* = 0.032), supporting the validity of the genetic instruments. Notably, GLP‐1R agonists were also associated with a significantly lower risk of OSA (OR = 0.83, 95% CI: 0.76 to 0.91, *p* = 6.15 × 10^−5^). No evidence of heterogeneity or horizontal pleiotropy was detected, and leave‐one‐out analysis confirmed the robustness of the findings.

**Conclusion:**

This study provides genetic evidence supporting the protective role of GLP‐1R agonists against OSA, highlighting their potential as a therapeutic strategy for OSA management.

## 1 Introduction

Obstructive sleep apnea (OSA) is a prevalent sleep‐related breathing disorder characterized by recurrent upper airway obstruction during sleep, resulting in daytime sleepiness, intermittent hypoxemia, hypercapnia, and sleep fragmentation. These disruptions impair glucose and lipid metabolism, cardiovascular function, and cognitive performance, contributing to comorbidities such as obesity, type 2 diabetes mellitus (T2DM), hypertension, heart failure, and neurocognitive decline [1]. OSA significantly reduces quality of life and poses serious health risks [1]. Affecting nearly one billion adults aged 30–69 years globally—with the highest prevalence reported in China—it represents an escalating public health challenge [2].

Current clinical management of OSA includes positional therapy, oral appliances, continuous positive airway pressure (CPAP), and upper airway surgery [1]. However, these approaches are often limited by poor adherence, intolerance, high costs, and restricted medical resources [3, 4]. CPAP, the standard of care, effectively reduces daytime sleepiness and improves quality of life. Nevertheless, long‐term adherence remains suboptimal, with only 60%–70% of patients maintaining consistent use [1]. Furthermore, CPAP has been associated with weight gain in individuals with OSA, raising concerns about its long‐term sustainability [5].

Obesity is the most significant modifiable risk factor for OSA, with the majority of affected individuals being overweight or obese [6]. Numerous studies have reported a strong positive correlation between body weight and OSA severity, commonly measured using the apnea‐hypopnea index (AHI). Each 1‐point increase in body mass index (BMI) is associated with a 14% increase in AHI, while a 0.1 increment in waist‐to‐hip ratio corresponds to an approximate 61% increase [7]. Additionally, a 10% gain in body weight elevates AHI by about 32%, whereas a 10% weight loss can reduce it by approximately 26% [8]. Obesity contributes to OSA pathogenesis through fat deposition around the upper airway [9, 10], reduced lung volume due to abdominal fat [11], and impaired respiratory control linked to leptin resistance [12]. Weight loss can significantly mitigate OSA severity by reversing these physiological alterations [6]. Furthermore, OSA may exacerbate obesity, establishing a bidirectional relationship [5]. Thus, weight management is a crucial component of OSA treatment in individuals with obesity.

Weight reduction strategies for OSA primarily include lifestyle and behavioral interventions, such as dietary modification and increased physical activity. Clinical trials have shown that weight loss through lifestyle changes can significantly improve AHI and alleviate OSA symptoms and associated comorbidities [9, 13, 14]. For patients who struggle to achieve sufficient weight loss through these approaches, anti‐obesity medications—such as phentermine/topiramate, liraglutide, and tirzepatide—have demonstrated efficacy in reducing OSA severity [15, 16, 17, 18]. Additionally, metabolic and bariatric surgeries (e.g., sleeve gastrectomy, Roux‐en‐Y gastric bypass, and gastric banding) have been shown to significantly lower AHI and improve OSA severity [9, 19, 20, 21, 22]. Despite these options, long‐term adherence to lifestyle modification remains challenging, and surgical interventions are invasive, limiting their widespread applicability. In this context, pharmacological treatments offer a promising alternative. However, no medication is currently approved specifically for the treatment of OSA in individuals with obesity, underscoring the urgent need for effective therapeutic agents.

Recent advances in pharmacotherapy—particularly with glucagon‐like peptide‐1 receptor (GLP‐1R) agonists (e.g., semaglutide) and GLP‐1/glucose‐dependent insulinotropic polypeptide (GIP) dual agonists (e.g., tirzepatide)—have significantly reshaped obesity management. Originally developed for glycemic control in T2DM, these agents have demonstrated substantial weight reducing effects [23, 24]. In addition to improving glycemic control, they enhance insulin sensitivity, exert anti‐inflammatory effects, and provide neuroprotective benefits [25]. These mechanisms may counteract key pathophysiological drivers of OSA, potentially reducing the treatment burden and healthcare costs. However, the effect of GLP‐1R agonists on OSA remains unclear. Although several randomized controlled trials (RCTs) have examined their impact on AHI, their findings have been inconsistent [16, 17, 26].

Mendelian randomization (MR) provides a robust approach to assess relationships by utilizing single‐nucleotide polymorphisms (SNPs) as instrumental variables (IVs) to estimate the effect of exposures on disease outcomes [27]. To date, no MR study has specifically evaluated the association between GLP‐1R agonists and OSA. This study aimed to address that gap by conducting an MR analysis to determine whether GLP‐1R agonist exposure is associated with OSA risk.

## 2 Methods

### 2.1 Genetic Instruments for GLP‐1R Agonists

**FIGURE 1:**
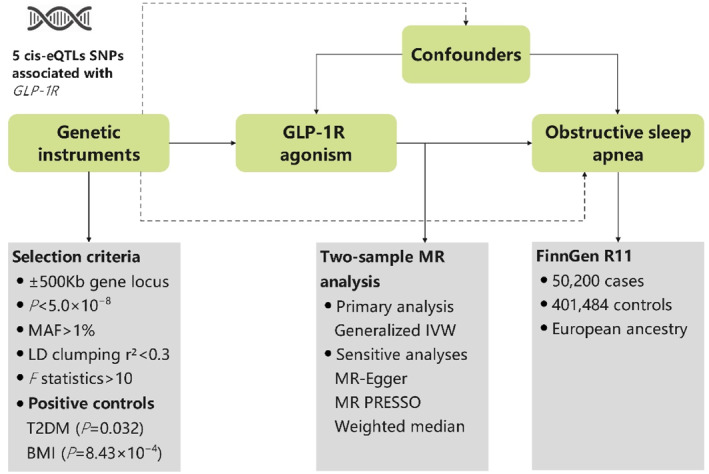
Study design outlining the selection of instrumental variables for genetically proxied GLP‐1R agonist exposure and subsequent MR analysis of OSA.

This study adheres to the STROBE‐MR (Strengthening the Reporting of Observational Studies in Epidemiology using Mendelian Randomization) guidelines for MR studies [28]. The study design is outlined in Figure 1 and involves four primary steps for selecting IVs representing GLP‐1R agonist exposure. First, *cis*‐expression quantitative trait loci (cis‐eQTLs) within 500 kb of the *GLP1R* gene were identified using data from the eQTLGen Consortium [29], serving as genetic proxies for GLP‐1R agonist exposure. Second, SNPs significantly associated with *GLP1R* expression in whole blood (*p* < 5.0 × 10^−8^) and with a minor allele frequency (MAF) > 1% were selected as candidate IVs. Third, to minimize linkage disequilibrium, a standard clumping procedure was applied using a correlation threshold of *r* ^2^ < 0.3. Finally, as GLP‐1R agonists are indicated for both glycemic control and weight loss, the validity of the selected genetic instruments was further assessed by testing their associations with T2DM and BMI.

### 2.2 Outcome Sources

To reduce population stratification bias, all analyses were restricted to individuals of European ancestry. BMI data were obtained from a genome‐wide association study (GWAS) of 681,275 European participants [30]. Summary statistics for T2DM were sourced from Xue et al. [31], encompassing 655,666 individuals of similar ancestry.

OSA data were retrieved from the FinnGen Release 11 (R11) cohort, which included 50,200 clinically diagnosed OSA cases and 401,484 controls of European descent. OSA diagnoses were based on clinical evaluation, self‐reported symptoms, and sleep registry data, including criteria such as a respiratory event index ≥ 5/h or an AHI ≥ 5/h [32]. A detailed summary of all datasets used is provided in Table S1.

### 2.3 Statistical Analyses

IVW method was employed as the primary analytical approach due to its precision and statistical efficiency [33]. The weighted median estimator was applied as a complementary method to assess the robustness of the results. Due to its power, the MR‐Egger method was primarily used to evaluate the direction and magnitude of potential bias rather than to determine statistical significance [34]. To assess instrument strength, *F* statistics were calculated, with values > 10 considered sufficient to reduce weak instrument bias. Sensitivity analyses included Cochran's Q test to evaluate heterogeneity; the MR‐Egger intercept and the Mendelian Randomization Pleiotropy RESidual Sum and Outlier (MR‐PRESSO) global test to detect horizontal pleiotropy; and leave‐one‐out (LOO) analysis to examine the influence of individual SNPs on the estimates. All statistical analyses were performed using the TwoSampleMR R package in R (version 4.4.0).

## 3 Results

### 3.1 Selection of Genetic Instruments

Five SNPs serving as proxies for GLP‐1R agonists are listed in Table S2. All IVs exhibited *F* statistics > 10, confirming their strength and reliability. Positive control analyses supported the validity of these instruments, showing significant associations with lower BMI (*β* = −0.063; 95% confidence interval [CI]: −0.10 to −0.03; *p* = 8.43 × 10^−4^) and reduced risk of T2DM (odds ratio [OR] = 0.80; 95% CI: 0.65 to 0.98; *p* = 0.032).

### 3.2 Effect of GLP‐1R Agonists on OSA

**FIGURE 2:**
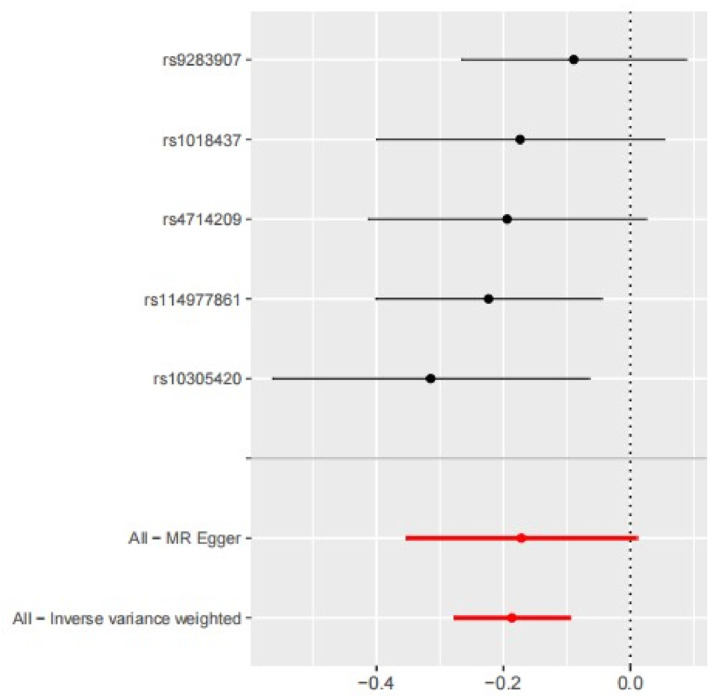
Forest plot of each SNP on OSA.

**FIGURE 3:**
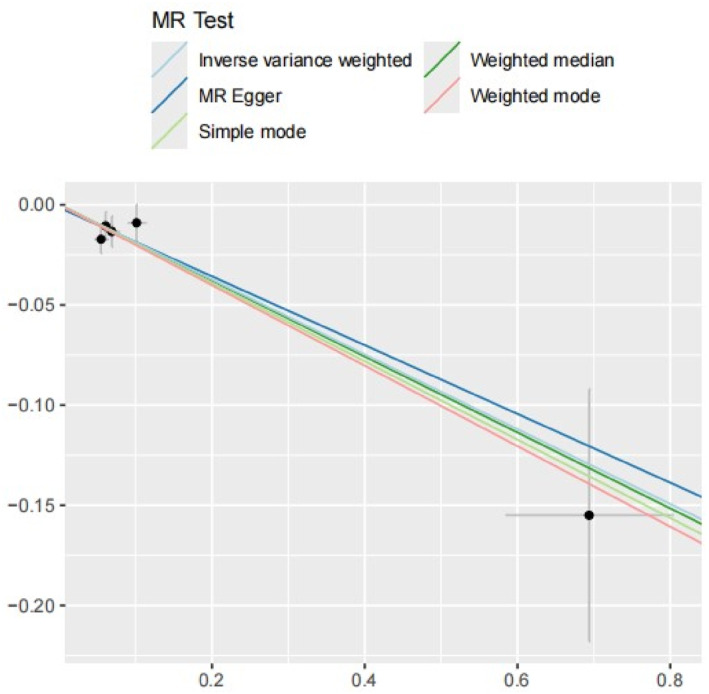
Scatter plot to depict the effect of genetically proxied GLP‐1R agonists on OSA.

**FIGURE 4:**
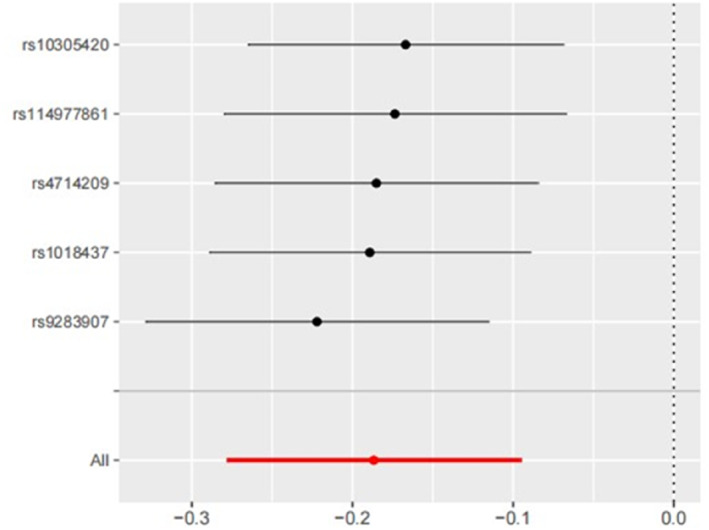
LOO analysis for the estimation of GLP‐1R agonists on OSA.

IVW analysis revealed a significant association between genetically proxied GLP‐1R agonist exposure and decreased risk of OSA (OR = 0.83; 95% CI: 0.76 to 0.91; *p* = 6.15 × 10^−5^) (Figures 2 and 3). This association was corroborated by the weighted median analysis but was not replicated using the MR‐Egger method, likely due to limited statistical power. No evidence of horizontal pleiotropy was detected using the MR‐Egger intercept and MR‐PRESSO global test (Table 1). Additionally, Cochran's Q test indicated no significant heterogeneity (*p* = 0.671) among the IVs, and the LOO analysis confirmed the robustness of the findings (Figure 4).

**TABLE 1:** MR analyses of genetically proxied GLP‐1R agonists on OSA.

Outcome	Method	OR (95% CI)	*P*	P*	*P* ^†^	*P* ^‡^
	IVW	0.83 (0.76–0.91)	6.15 × 10^−5^	0.671		
Obstructive	MR‐Egger	0.84 (0.70–1.01)	0.161	0.509	0.863	
Sleep apnea	MR‐PRESSO	0.83 (0.77–0.89)	6.42 × 10^−3^			0.715
	Weighted median	0.83 (0.74–0.93)	1.06 × 10^−3^			

Note: P*, *p*‐value for heterogeneity test; *P* ^†^, *p*‐value for MR‐Egger intercept; *P* ^‡^, *p*‐value for the global test.

## 4 Discussion

This MR analysis identified an inverse association between GLP1R gene expression and the risk of OSA, suggesting that pharmacologic activation of the GLP‐1R—as achieved by GLP‐1R agonist use—may reduce OSA risk. The validity of our IVs was supported by strong associations with established therapeutic effects of GLP‐1R agonists, including reductions in BMI and risk of T2DM. These positive control results enhance confidence in the observed inverse association between GLP‐1R agonist exposure and OSA. To our knowledge, this is the first MR study to demonstrate a significant relationship between GLP‐1R agonists and OSA.

Clinical trials investigating the therapeutic efficacy of GLP‐1R agonists in OSA remain limited and have reported inconsistent findings. Several studies have reported that GLP‐1R agonists—particularly liraglutide—either alone or in combination with CPAP, can significantly reduce the AHI or excessive daytime sleepiness [16, 17, 35]. Our findings align with these results, reinforcing the hypothesis that GLP‐1R agonists may mitigate OSA severity through metabolic improvements, including weight loss and glucose control. One RCT indicated that CPAP alone or in combination with liraglutide over 24 weeks was more effective at reducing AHI than liraglutide alone [26]. Notably, liraglutide did not yield a statistically significant improvement in AHI over baseline at 24 weeks [26]. These discrepancies may stem from variations in study design, small sample sizes, limited durations, and confounding factors—issues that inherently limit observational and interventional studies. The MR study design, which represents a lifelong genetic modulation of GLP‐1R agonist's targets, may partially compensate for these deficiencies.

Moreover, existing clinical studies have focused primarily on liraglutide, while the effects of other GLP‐1R agonists—such as dulaglutide and semaglutide—remain largely unexamined in the context of OSA. Our MR results suggest a potential class effect of GLP‐1R agonists in reducing OSA risk. This notion is further supported by recent findings from the SURMOUNT‐OSA trial, which reported that tirzepatide—a dual GLP‐1/GIP receptor agonist—significantly improved OSA severity alongside weight loss and cardiometabolic benefits [18]. Collectively, these findings highlight the promise of incretin‐based therapies in OSA management, particularly in patients with comorbid obesity, T2DM, and OSA, due to their metabolic benefits.

Although the precise mechanisms by which GLP‐1R agonists exert protective effects against OSA remain to be fully elucidated, several plausible pathways have been proposed: (1) Weight reduction: Obesity is a key etiological factor in OSA, contributing to its pathogenesis through fat deposition around the upper airway [9, 10], reduced lung volume due to abdominal fat [11], and impaired respiratory control linked to leptin resistance [12]. GLP‐1R agonists promote weight loss, and reductions in BMI, body weight, and waist circumference are strongly correlated with improvements in AHI and daytime somnolence [16, 17, 35]. (2) Metabolic regulation: OSA and T2DM are pathophysiologically intertwined. GLP‐1R agonists improve insulin sensitivity, glycemic control, and metabolic flexibility [1]. (3) Central nervous system effects: GLP‐1 receptors are expressed in brain regions implicated in respiratory regulation. Their activation may enhance central respiratory drive, reduce breathing instability, and modulate ventilatory responses during sleep [36]. (4) Neuroprotection: GLP‐1R agonists exhibit a spectrum of neuroprotective effects—including promoting neuronal survival, reducing oxidative stress, mitigating neuroinflammation, and enhancing neurogenesis [25, 36]. These protective mechanisms may help prevent neurocognitive impairments associated with OSA, as evidenced by studies in T2DM [37], Parkinson's disease, and Alzheimer's disease [38].

This study has several limitations. First, the genetic analysis was conducted primarily in individuals of European ancestry, which may limit the generalizability of the findings to other ethnic groups. Second, interindividual pharmacokinetics, including differences in drug metabolism, dosage, and specific formulations of GLP‐1R agonists, were not captured, which may influence treatment effects. Third, the MR estimates reflect the effects of lifelong genetically proxied GLP‐1R activation; thus, short‐term pharmacologic outcomes may differ and should be evaluated in clinical settings.

Given the genetic evidence demonstrated in our study, future clinical trials should assess the efficacy and safety of GLP‐1R agonists specifically for OSA treatment, including in non‐obese individuals and across diverse populations. If validated, these findings could inform updates to clinical practice guidelines, positioning GLP‐1R agonists as adjunctive or alternative therapies to CPAP, particularly in patients with metabolic comorbidities. Furthermore, integrating metabolic therapies into OSA management could represent a paradigm shift toward a more holistic and personalized treatment strategy, ultimately improving long‐term metabolic outcomes in patients with OSA.

## 5 Conclusion

This MR study provides genetic evidence supporting an inverse association between GLP‐1R agonist use and the risk of OSA. These findings suggest that GLP‐1R agonists may represent a novel therapeutic approach for OSA management, particularly in patients with coexisting metabolic disorders. However, further clinical validation and mechanistic studies are needed to fully elucidate their role and optimize their integration into OSA treatment strategies.

## Author Contributions

Conceptualization: Q.B.B. data analysis and initial drafting of the manuscript: Q.B.B. and H.Y.J. (These two authors should be regarded as co‐first authors) revision and reviewing of the manuscript at all stages preparation: Y.C.F. revising the paper: F.S.Y. and S.D.M.

## Ethics Statement

This study utilized data from publicly available databases, with ethical approval previously granted in the original studies.

## Conflicts of Interest

The authors declare no conflicts of interest.

## Supporting information


**Table S1**: Dataset description.

**Table S2**: The genetic variants selected for the Instrument Variables (IVs) of GLP‐1R agonists.

## Data Availability

All relevant data are included within the manuscript and its supplementary materials.
