# Supplementary material for: Effect of Glucagon‐Like Peptide 1 Receptor Agonists on Obstructive Sleep Apnea
Source: Obes Sci Pract. 2025 Aug 22;11(4):e70090. doi: 10.1002/osp4.70090 (PMC12371556; doi:10.1002/osp4.70090)
Supplement: Supplementary file 1 — Table S1: Dataset description. [file OSP4-11-e70090-s001.docx]

| **Supplementary Table 1. Dataset description** | | | | | | |
| --- | --- | --- | --- | --- | --- | --- |
| **Phenotype** | **Consortium** | **GWAS ID** | **Sample size** | **Ancestry** | **PMID** | **Data source** |
| Type 2 diabetes mellitus | DIAGRAM, GERA, UK Biobank | GCST006867 | 655,666 | European | 30054458 | https://gwas.mrcieu.ac.uk/datasets/ebi-a-GCST006867/ |
| Body mass index | GIANT | ieu-b-40 | 681,275 | European | 30124842 | https://gwas.mrcieu.ac.uk/datasets/ieu-b-40/ |
| Obstructive sleep apnea | FinnGen | NA | 451,684 | European | NA | https://r11.finngen.fi/pheno/G6_SLEEPAPNO |
| Abbreviations: DIAGRAM, Diabetes Genetics Replication and Meta-analysis; GERA, Genetic Epidemiology Research on Aging; GIANT, Genetic Investigation of Anthropometric Traits; GWAS, genome-wide association study. | | | | | | |
