# Supplementary material for: Effect of Glucagon‐Like Peptide 1 Receptor Agonists on Obstructive Sleep Apnea
Source: Obes Sci Pract. 2025 Aug 22;11(4):e70090. doi: 10.1002/osp4.70090 (PMC12371556; doi:10.1002/osp4.70090)
Supplement: Supplementary file 2 — Table S2: The genetic variants selected for the Instrument Variables (IVs) of GLP‐1R agonists. [file OSP4-11-e70090-s002.docx]

| **Supplementary Table 2. The genetic variants selected for the Instrument Variables (IVs) of GLP-1R agonists** | | | | | | | | | |
| --- | --- | --- | --- | --- | --- | --- | --- | --- | --- |
| **SNP** | **Gene** | **Effect allele/**  **Non-effect allele** | **Effect allele frequency** | **Beta** | **Se** | **P** | **Sample size** | **R^2^-variance explained** | **F-statistics** |
| rs1018437 | GLP-1R | C/T | 0.437 | -0.061 | 0.008 | 2.02E-13 | 29621 | 1.82E-03 | 53.99 |
| rs10305420 | GLP-1R | T/C | 0.364 | 0.055 | 0.009 | 1.95E-10 | 29085 | 1.39E-03 | 40.52 |
| rs114977861 | GLP-1R | C/T | 0.013 | 0.694 | 0.109 | 2.07E-10 | 3243 | 1.23E-02 | 40.40 |
| rs4714209 | GLP-1R | T/C | 0.253 | 0.069 | 0.009 | 4.18E-13 | 29294 | 1.79E-03 | 52.56 |
| rs9283907 | GLP-1R | A/G | 0.151 | 0.101 | 0.012 | 1.52E-18 | 29294 | 2.63E-03 | 77.23 |
